# Hemodynamic Assessment of Arterial Perfusion by Using the Ultrasonographic Hand Acceleration Time: Protocol for a Cross-Sectional Prospective Descriptive Study

**DOI:** 10.2196/64450

**Published:** 2025-08-21

**Authors:** Begoña Gonzalo, Sebastián Videla, José Moranas, Thiago Carnaval, Carolina Herranz, Emma Espinar, Elena Iborra

**Affiliations:** 1 Vascular and Endovascular Surgery Department Bellvitge University Hospital Barcelona Spain; 2 Clinical Pharmacology Department Hospital Universitari Germans Trias i Pujol Badalona Spain; 3 Pharmacology Unit, Department of Pathology and Experimental Therapeutics School of Medicine and Health Sciences Universitat de Barcelona Barcelona Spain; 4 Design and Biometrics Department Medicxact Madrid Spain; 5 Oncology Data Analytics Program Institut Català d'Oncologia L'Hospitalet de Llobregat Spain

**Keywords:** hand arterial perfusion, hand acceleration time, HAT, acceleration time, chronic upper limb ischemia, duplex ultrasound, arterial, acceleration, upper limb, protocol, cross-sectional study, ultrasound, ischemia, hand, arteries, descriptive analysis, ultrasonographic

## Abstract

**Background:**

Several noninvasive methods can be used to assess hand perfusion, but none have been standardized for diagnosing chronic upper limb ischemia. One potentially useful diagnostic tool for this purpose is the ultrasonographic hand acceleration time (HAT), although it still requires characterization and validation.

**Objective:**

This study aims to describe the technique for assessing hand perfusion through HAT while quantifying it in patients with chronic upper limb ischemia and healthy volunteers.

**Methods:**

This prospective, cross-sectional, and descriptive study evaluates HAT measurements by using vascular duplex ultrasound (DUS), a novel technique for assessing hand arterial perfusion. This study includes adult patients with chronic upper limb ischemia and healthy volunteers without cardiovascular risk factors. HAT was measured in 4 different hand arteries. A standard descriptive analysis was performed, along with comparisons using the Mann-Whitney *U* test, when applicable. This study received institutional review board approval, and no study-related procedures were performed prior to signing the written informed consent. This research complied with the Declaration of Helsinki, Good Clinical Practice guidelines, and Spanish regulatory requirements.

**Results:**

As of June 2023, 30 participants were enrolled in the study, comprising 15 patients and 15 healthy volunteers, resulting in a total of 15 ischemic and 30 nonischemic hands. Data analyses were concluded in late November 2023, and results were sent for publication in February 2024. The manuscript containing the results was accepted in May 2024 and published online in July 2024, becoming available in PubMed in December 2024. The results have already been made available to the scientific community prior to the submission of this protocol. This study protocol was submitted for publication in July 2024 to enhance transparency and to offer comprehensive methodological insight into the HAT technique. No additional analyses beyond those reported in the published study are planned.

**Conclusions:**

This study presents the first systematic evaluation of HAT measurements by using vascular DUS as a novel diagnostic tool for chronic upper limb ischemia. By comparing ischemic and nonischemic hands, this study aims to establish preliminary cutoff values that may aid clinical decision-making. Although prior studies have only reported HAT measurements in case reports, this study provides quantitative data on its feasibility and potential clinical application. Despite its small sample size, which limits subgroup analysis and generalizability, the study serves as a foundation for future research by providing essential data for refining cutoff values and informing sample size calculations for larger studies. Although examiner dependence could introduce systematic bias, the standardized methodology ensures consistency across all measurements. Ultimately, HAT may emerge as a quick, noninvasive diagnostic tool for chronic upper limb ischemia, potentially complementing existing nonstandardized methods such as the digital-brachial index, digital pressures, and plethysmography.

**Trial Registration:**

ClinicalTrials.gov NCT05977725; https://clinicaltrials.gov/study/NCT05977725

**International Registered Report Identifier (IRRID):**

RR1-10.2196/64450

## Introduction

Different noninvasive techniques can be used for evaluating arterial perfusion in the hand, which include measuring the digital-brachial index (DBI), digital arterial pressures, oxygen saturation, and plethysmographic signals [1]. However, despite their availability, none have become the accepted standard for diagnosing chronic ischemia of the upper extremities, likely due to the rarity and etiological diversity of this condition.

In contrast to its lower limb counterpart [2], upper limb vascular diseases (whether acute or chronic) represent <10% of the consultations in vascular surgery settings [1]. Among these, only a minority (around 4%) ultimately require revascularization procedures [3]. The underlying causes of upper extremity ischemia are varied and include atherosclerosis, thoracic outlet syndrome, different forms of vasculitis (such as Takayasu arteritis, giant cell arteritis, and Buerger disease), connective tissue disorders, trauma, unresolved thrombotic events [3,4], and ischemia secondary to vascular access in patients undergoing dialysis [5].

Particularly noteworthy is hemodialysis access–induced distal ischemia (HAIDI), which affects up to 5%-7% of individuals with end-stage renal disease undergoing dialysis via an arteriovenous access [6]. Although chronic upper limb ischemia is rare overall, HAIDI represents a considerable subset within this population. DBI and digital pressures have been investigated extensively in this context, but no clear consensus exists on their diagnostic thresholds or reliability [7-9]. Furthermore, although adaptations of Fontaine’s classification have been proposed for UPPER limb ischemia, corresponding objective testing metrics such as DBI or digital pressure stratification have not been standardized as they have for lower limb disease.

Because of these limitations, imaging modalities such as computed tomography angiography, magnetic resonance angiography, or arteriography are often needed to confirm a diagnosis of upper limb arterial occlusive disease [3,10]. On the other hand, duplex ultrasound (DUS) is a noninvasive, dynamic tool that can visualize the entire upper extremity arterial tree, and prior studies have successfully described the anatomical characteristics of the hand vasculature by using this approach [11].

One specific DUS parameter—acceleration time—has emerged as a potential indicator of distal arterial compromise. Acceleration time quantifies the interval in milliseconds between the onset of the systole and the systolic peak on Doppler waveform analysis, providing real-time insight into arterial compliance and resistance [12]. The diagnostic applicability of acceleration time has been established in different vascular beds, particularly in the pedal arteries for assessing lower limb ischemia (pedal acceleration time) [13-15]. Additionally, acceleration time has been studied in arterial territories such as the carotid arteries [16], pulmonary arteries [17], and the aorta [18]. Some authors have applied this concept to the upper extremity, measuring what is referred to as the hand acceleration time (HAT) [19-21]. However, current data are limited, mostly consisting of small case series or anecdotal reports, and no formal characterization or validation of HAT has been conducted to date.

In light of this, our study aims to describe a reproducible technique for measuring HAT by using DUS and to assess its diagnostic potential by comparing patients with chronic upper limb ischemia to healthy control participants. We hypothesized that HAT is a useful diagnostic tool for chronic upper limb ischemia, considering that ischemic hands would exhibit prolonged acceleration time values due to impaired perfusion, which would broaden the arterial spectral Doppler waveform. This study aims to describe the technique for assessing hand perfusion through HAT while quantifying it in patients with chronic upper limb ischemia and healthy volunteers.

## Methods

### Study Design

This was a cross-sectional, prospective, and descriptive study of HAT measurements—a hemodynamic assessment technique—with vascular DUS for assessing the hand’s arterial perfusion. Notably, vascular DUS with HAT measurements is a new technique that has not been previously described.

### Study Population and Setting

The study population consisted of patients with known chronic upper limb ischemia who were observed in the Angiology and Vascular Surgery outpatient clinic or hospitalized in the Vascular Surgery Unit, and they served as the exposed population. In the diseased cohort, we classified chronic upper limb ischemia by extrapolating Fontaine’s clinical classification of chronic lower limb ischemia [22]: (1) grade I: asymptomatic, (2) grade II: arm claudication, (3) grade III: rest pain, and (4) grade IV: trophic lesions.

The diagnosis of chronic upper limb ischemia in the study participants was primarily clinical based on the presence of characteristic signs and symptoms, including the absence of upper extremity pulses and presence of arm claudication, rest pain, or trophic lesions. Additionally, although we assessed digital pressure reductions in all patients, these measurements were not included in this study due to the lack of standardized DBI criteria for diagnosing chronic upper limb ischemia. To confirm the diagnosis and evaluate arterial involvement, DUS was performed to identify stenotic or occlusive lesions. DBI and digital pressures were not used for clinical staging since they have not been standardized for the upper limb arterial disease and are not a gold standard for comparison. We invited the patients’ escorts to participate in the study as healthy volunteers (who served as the unexposed population for comparison purposes). All study-related procedures were performed at the Bellvitge University Hospital.

### Eligibility Criteria

Textbox 1 summarizes the eligibility criteria for patients with chronic upper limb ischemia and healthy volunteers. Both must meet all the inclusion criteria and none of the exclusion criteria to participate in the study.

**Eligibility criteria.**

**Inclusion criteria**

- Patients with upper extremity chronic ischemia
  Adult (≥18 years) patients
  Either biological sex
  Diagnosed with chronic ischemia of the upper extremity
  Who sign the written informed consent

- Healthy volunteers
  Healthy adult (≥18 years of age) volunteers
  Either biological sex
  Who sign the written informed consent

**Exclusion criteria**

- Patients with upper extremity chronic ischemia
  Patients deemed unable to understand the study by the investigator

- Healthy volunteers
  Healthy volunteers deemed unable to understand the study by the investigator
  Healthy volunteers presenting any known cardiovascular risk factor (eg, smoking habit, arterial hypertension, diabetes mellitus, dyslipidemia)

### Study Procedures

All study participants (patients with chronic upper limb ischemia and healthy volunteers) underwent hand DUS at the Bellvitge University Hospital. Participants were instructed to rest in a supine position for a minimum of 10 minutes prior to the ultrasound examination to ensure hemodynamic stability; the environment temperature was set at approximately 22 °C. A clinical assessment was conducted before imaging, with particular attention to distal pulse palpation in the upper limbs. Ultrasound assessments were performed with EPIQ Elite (Philips Healthcare), utilizing a high-resolution linear transducer operating at 22 MHz to measure hemodynamic parameters.

**Figure 1:**
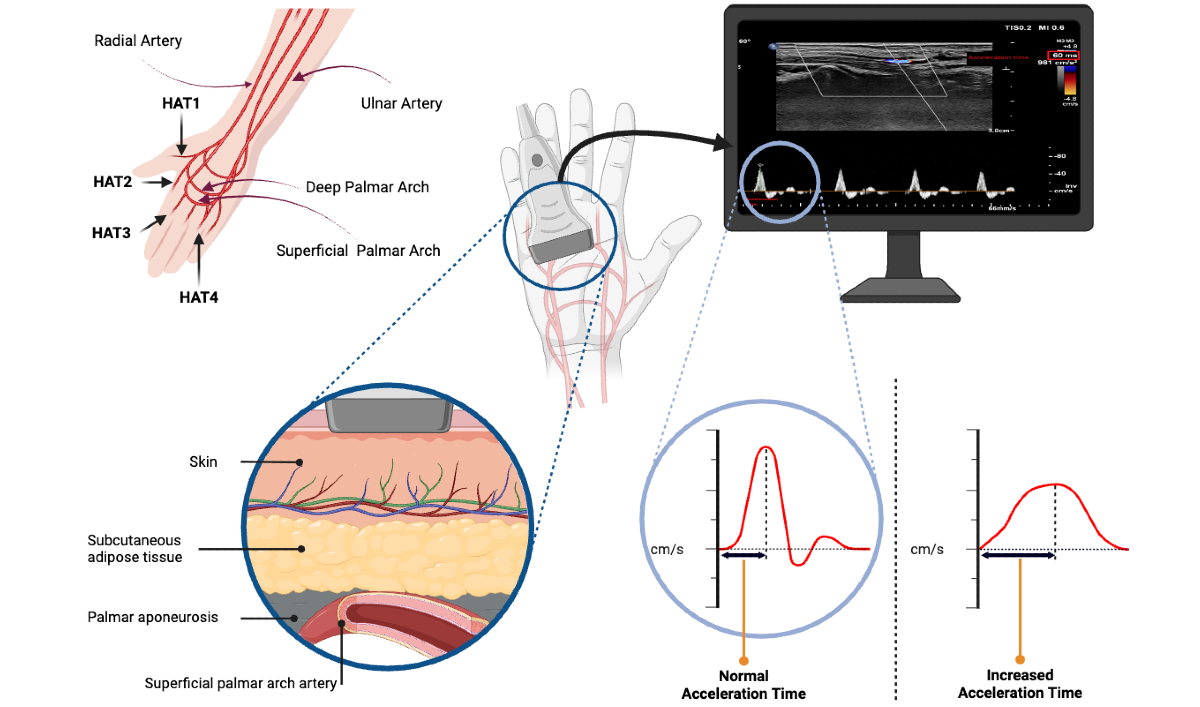
A normal HAT occurs in the absence of upper limb ischemia (with a narrow triphasic spectral waveform), while an increased HAT arises in the setting of upper limb ischemia (with a decreased systolic up-rise and wide waveform). HAT: hand acceleration time.

Optimizing ultrasound settings (such as increasing color gain and lowering pulse repetition frequency) is crucial for accurate visualization of small-caliber hand arteries. After locating the artery, the Doppler “acceleration time or time/slope” function was used to assess HAT after adjusting the spectral waveform in the spectral window and measuring the interval from the onset of systolic flow to its peak velocity (Figure 1). In healthy arteries, the waveform typically exhibits a triphasic pattern with a steep upstroke and short acceleration time, whereas in ischemic vessels, the waveform broadens and the acceleration is delayed.

**Figure 2:**
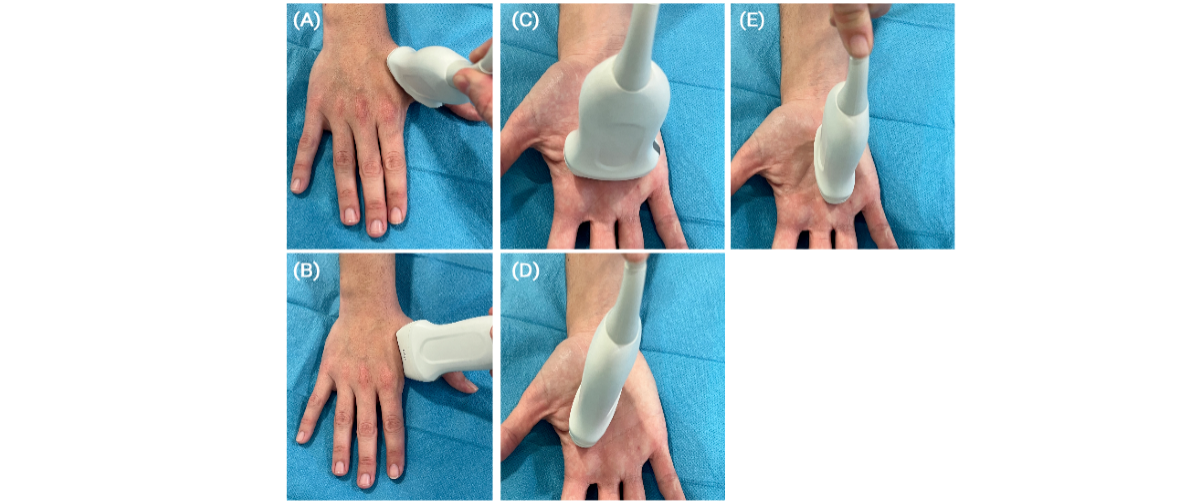
HAT measuring sites. (A) HAT1 measuring site, (B) HAT2 measuring site, (C) HAT3 and HAT4 measuring sites (transversal view), (D) HAT3 measuring site (longitudinal view), and (E) HAT4 measuring site (longitudinal view). Of note, HAT3 and HAT4 are both measured twice with the same views. HAT: hand acceleration time.

Considering the anatomical variability in the hand’s arterial vascularization, we measured HAT in 4 hand arteries and the distal ulnar and radial arteries. HAT1 was measured at the princeps pollicis artery by placing the transducer on the dorsal side of the hand at the level of the first finger (Figure 2A). The radial artery in the hand lies in the anatomical snuffbox between the extensor pollicis longus and extensor pollicis brevis muscles. The princeps pollicis artery arises from this point of the radial artery as it runs ulnarward and dorsalward to the extensor pollicis longus. Similarly, HAT2 was assessed by keeping the hand at the same position and placing the transducer on the first commissure on the volar metacarpal surface (Figure 2B)—it was measured at the radial artery of the index finger. HAT3 and HAT4 were measured at the first and third common palmar digital arteries, respectively, through the hand’s palmar surface. First, the transducer was transversally over the metacarpal head and distal to the hand fold (Figure 2C). After locating the common palmar digital artery between the extensor muscles and above the metacarpus, we located the common palmar digital artery and measured HAT3 at the first common palmar digital artery and HAT4 at the third common palmar digital artery. Thereafter, the transducer was positioned in a longitudinal view (Figure 2D), and we repeated both HAT3 and HAT4 assessments.

We performed 3 measurements per artery to minimize variability and to ensure consistency during the evaluation.

### Outcomes

Our primary variables were HAT measurements (in milliseconds) with DUS in the (1) princeps pollicis artery (HAT1), (2) radialis indicis artery (radial artery of the index finger; HAT2), (3) first common digital artery (HAT3), and (4) third common digital artery (HAT4) (Figure 1).

The secondary variables included (1) the absolute and relative frequencies of baseline and demographic characteristics (age and biological sex), (2) the cumulative incidence of cardiovascular risk factors (eg, smoking habit, arterial hypertension, diabetes, dyslipidemia), (3) the cumulative incidence of diseases of interest (ie, end-stage renal disease, ischemic heart disease, heart failure, chronic obstructive pulmonary disease), (4) the absolute and relative frequencies of each etiology of ischemic upper limb disease (eg, subclavian, humeral, distal stenosis/occlusion, end-stage renal disease-secondary lesions), (5) the clinical stage of upper limb disease (eg, asymptomatic, arm claudication, trophic lesions), and (6) the presence of distal pulses in the upper limb in the physical examination.

### Ethical Considerations

This study was approved by the Bellvitge University Hospital’s institutional review board (registration ICP013/23) in May 2023. All participants provided written informed consent in accordance with the updated Declaration of Helsinki, Good Clinical Practice guidelines, and applicable Spanish and European regulatory requirements prior to any study procedures. Participation was voluntary, and participants were informed that they could withdraw from the study at any time without providing a reason and without any consequences. All collected data were deidentified prior to analysis, ensuring that no individual could be identified in the published results. Confidentiality was safeguarded in compliance with the current Spanish data protection law (LOPD 3/2018) and the European General Data Protection Regulation (EU regulation 2016/679 of the European Parliament and Council of April 27, 2016). Notably, this publication strictly reflects the version approved by the institutional review board and was not modified either before or after the study results were published elsewhere [23]. Participants, including both patients and healthy volunteers, did not receive any financial or nonfinancial compensation for their participation. This study was prospectively registered on ClinicalTrials.gov (NCT05977725) and adheres to the STROBE (Strengthening the Reporting of Observational studies in Epidemiology) guidelines for reporting observational studies.

### Data Sources, Data Collection, and Quality Control

Our data sources were clinical interviews, individual medical histories, and sonographic examinations performed on all study participants. All study-related information was registered in the participant’s medical history and in an ad hoc–created database. This database was made up of anonymized data, and only the principal investigator and authorized study team members had access to it. All data were dissociated, and participants were assigned a number (code) upon enrollment. To ensure data accuracy, the principal investigator reviewed all individual medical histories as a quality control measure.

### Sample Size and Statistical Analysis

Given the exploratory nature of this study, we did not perform a formal sample size calculation. Considering the aforementioned disease’s low prevalence, we intended to include all 15 patients diagnosed with chronic upper limb ischemia and observed in our center’s outpatient clinic, as well as the 30 hands of 15 healthy volunteers. We performed a standard descriptive analysis of all the study variables. We summarized categorical variables as absolute and relative frequencies and continuous variables as medians and range. The nonparametric Mann-Whitney *U* test (independent samples) was used for between-group comparisons regarding HAT values in 4 digital locations (princeps pollicis artery, radialis indicis artery, first common digital artery, and third common digital artery) as well as in the distal radial and ulnar arteries [24]. Given the exploratory nature of this study, a formal sample size estimation was not conducted. Notably, we must underscore that this is a preliminary feasibility study with a sample size that is too small to allow analysis adjustments for more than one variable. All statistical analyses were performed with R 4.3 software or higher (R Foundation for Statistical Computing) for Windows.

## Results

As of June 2023, 30 participants were enrolled in the study, comprising 15 patients and 15 healthy volunteers, resulting in a total of 15 ischemic and 30 nonischemic hands. Data analyses were concluded in late November 2023, and results were sent for publication in February 2024. The manuscript containing the results was accepted in May 2024 and published online in July 2024, becoming available in PubMed in December 2024 [23]. This study protocol was submitted for publication in July 2024 to enhance transparency and offer comprehensive methodological insight into the HAT technique. No modifications were made to the original protocol used to guide the results, and no further analyses beyond those reported in the published study are planned.

## Discussion

This study protocol outlines the design and methodology of a cross-sectional, prospective, and descriptive investigation into HAT as a diagnostic marker for chronic upper limb ischemia. Although the sequence of the publication is atypical (ie, protocol being published after the dissemination of the corresponding results [23]), it reflects the intent to foster transparency and provide guidance for the future applications of this technique.

To date, the clinical application of HAT has been explored only in a handful of case reports. Notably, one series described its application in 4 patients with HAIDI and associated trophic lesions, where HAT values declined after revascularization, coinciding with symptom resolution [19]. In another case report, palmar DUS was used to guide the need for reperfusion in a patient with subclavian artery disruption and scapulothoracic dissociation, accurately assessing the function of an extra-anatomic bypass [20]. Finally, HAT was successfully used to detect early signs of posthemorrhagic shock state [21]. These preliminary findings suggest that HAT may hold promise as a rapid, noninvasive diagnostic tool for assessing upper limb perfusion and might potentially complement or even replace other noninvasive techniques such as DBI, digital pressures, and plethysmography (which are currently standardized in this context). Nonetheless, this will require future robust prospective studies addressing the sensitivity, specificity, and predictive values of HAT in comparison to these existing modalities.

Herein, we propose a structured and reproducible method for measuring HAT by using DUS in both patients with chronic upper limb ischemia and healthy volunteers. Although the study was not powered to establish definitive diagnostic thresholds, the reported ranges might serve as preliminary reference for future research. Given its descriptive nature and limited sample size, stratifying HAT values across different types of arterial disease was deemed impractical, as subgroup analysis would likely reduce (if not mislead) interpretability. However, this study does describe the anatomical location of the affected arteries, bearing in mind that HAT values tend to be elevated in ischemic hands regardless of the disease site when compared to healthy limbs.

Markedly, this study might incur information bias since the HAT results, like most sonographic measurements, are examiner-dependent; however, if it were the case, it would be a systematic bias (since the same examiner performed all the measurements), yielding similar probabilities of occurrence in all study participants. The small sample size does not allow analysis adjustments for more than one variable and acts as another limitation. Further, generalizing its results to a broader population requires caution, given these limitations; however, they may serve as guidance for conducting future large-scale studies.

Transcutaneous oxygen pressure (TcPO_2_) and skin perfusion pressure are valuable tools for assessing tissue perfusion, particularly in the context of lower limb ischemia, and exploring their correlation with HAT in cases of upper limb ischemia would be of interest. However, although TcPO_2_ has been well validated for lower limb assessments, no consensus currently exists regarding reference values or diagnostic thresholds for hand ischemia. Based on our clinical experience—despite having previously used TcPO_2_ routinely for lower limb evaluation—we found the test to be time-consuming, technically challenging for the upper extremities, and frequently inconclusive in guiding clinical decision-making. Consequently, we progressively discontinued its use in our daily practice. Given these limitations and the lack of validated upper-limb parameters, TcPO_2_ was not included in this study. Nevertheless, future research examining the correlation between HAT and alternative perfusion metrics such as TcPO_2_ or skin perfusion pressure may contribute to further validating HAT as a diagnostic tool for hand ischemia.

On the other hand, the rich collateral circulation of the hand—arising primarily from the radial, ulnar, and interosseous arteries—plays a significant role in modulating perfusion and may influence arterial Doppler waveforms, potentially affecting HAT results. The HAT protocol was intentionally designed to assess the overall arterial supply to the hand and to provide an indirect measure of collateral flow. To ensure consistency and reliability, we selected 4 arterial segments for evaluation: 2 branches from the radial artery and 2 from the ulnar artery. This choice was based on anatomical studies indicating relatively low interindividual variability in these segments, thereby supporting reproducibility across participants while adequately capturing the contributions of both major vascular axes.

## Abbreviations

DBI: digital-brachial index
DUS: duplex ultrasound
HAIDI: hemodialysis access–induced distal ischemia
HAT: hand acceleration time
STROBE: Strengthening the Reporting of Observational studies in Epidemiology
TcPO_2_: transcutaneous oxygen pressure

## Data Availability

Datasets used and/or analyzed during this study are available from the corresponding author upon reasonable request immediately after publication.

## References

[1] Deguara J, Ali T, Modarai B, Burnand KG (2005). Upper limb ischemia: 20 years experience from a single center. Vascular.

[2] Bae M, Chung SW, Lee CW, Choi J, Song S, Kim S (2015). Upper limb ischemia: clinical experiences of acute and chronic upper limb ischemia in a single center. Korean J Thorac Cardiovasc Surg.

[3] Spinelli F, Benedetto F, Passari G, La Spada M, Carella G, Stilo F, De Caridi G, Lentini S (2010). Bypass surgery for the treatment of upper limb chronic ischaemia. Eur J Vasc Endovasc Surg.

[4] Alef M, Hamdan A, Cronenwett JL, Johnston KW (2014). Upper extremity arterial disease: general considerations. Rutherford's Vascular Surgery, 2-Volume Set. 8th ed.

[5] Tordoir J H M, Dammers R, van der Sande F M (2004). Upper extremity ischemia and hemodialysis vascular access. Eur J Vasc Endovasc Surg.

[6] Schmidli J, Widmer MK, Basile C, de Donato G, Gallieni M, Gibbons CP, Haage P, Hamilton G, Hedin U, Kamper L, Lazarides MK, Lindsey B, Mestres G, Pegoraro M, Roy J, Setacci C, Shemesh D, Tordoir JHM, van Loon M, Kolh P, de Borst GJ, Chakfe N, Debus S, Hinchliffe R, Kakkos S, Koncar I, Lindholt J, Naylor R, Vega de Ceniga M, Vermassen F, Verzini F, Mohaupt M, Ricco J, Roca-Tey R, Esvs Guidelines Committee, Esvs Guidelines Reviewers (2018). Editor's Choice - Vascular Access: 2018 Clinical Practice Guidelines of the European Society for Vascular Surgery (ESVS). Eur J Vasc Endovasc Surg.

[7] Malik J, Tuka V, Kasalova Z, Chytilova E, Slavikova M, Clagett P, Davidson I, Dolmatch B, Nichols D, Gallieni M (2008). Understanding the dialysis access steal syndrome. A review of the etiologies, diagnosis, prevention and treatment strategies. J Vasc Access.

[8] Modaghegh MS, Kazemzadeh G, Pezeshki Rad M, Ravari H, Hafezi S, El-Husheimi A, Barzanouni A (2015). Chronic hemodialysis access-induced distal ischemia (HAIDI): distinctive form of a major complication. J Vasc Access.

[9] Oprea A, Molnar A, Scridon T, Mircea PA (2019). Digital pressure in haemodialysis patients with brachial arteriovenous fistula. Indian J Med Res.

[10] Aboyans V, Ricco J, Bartelink M, Björck M, Brodmann M, Cohner T, Collet J, Czerny M, De Carlo M, Debus S, Espinola-Klein C, Kahan T, Kownator S, Mazzolai L, Naylor R, Roffi M, Röther J, Sprynger M, Tendera M, Tepe G, Venermo M, Vlachopoulos C, Desormais I (2017). 2017 ESC Guidelines on the Diagnosis and Treatment of Peripheral Arterial Diseases, in collaboration with the European Society for Vascular Surgery (ESVS). Kardiol Pol.

[11] Langholz J, Ladleif M, Blank B, Heidrich H, Behrendt C (1997). Colour coded duplex sonography in ischemic finger artery disease--a comparison with hand arteriography. Vasa.

[12] Trihan J, Mahé G, Laroche J, Dauzat M, Perez-Martin A, Croquette M, Lanéelle D (2023). Arterial blood-flow acceleration time on doppler ultrasound waveforms: what are we talking about?. J Clin Med.

[13] Sommerset J, Karmy-Jones R, Dally M, Feliciano B, Vea Y, Teso D (2019). Plantar acceleration time: a novel technique to evaluate arterial flow to the foot. Ann Vasc Surg.

[14] Teso D, Sommerset J, Dally M, Feliciano B, Vea Y, Jones RK (2021). Pedal Acceleration Time (PAT): a novel predictor of limb salvage. Ann Vasc Surg.

[15] Trihan J, Mahé Guillaume, Croquette M, Coutant V, Thollot C, Guillaumat J, Lanéelle Damien (2021). Accuracy of acceleration time of distal arteries to diagnose severe peripheral arterial disease. Front Cardiovasc Med.

[16] Strosberg DS, Haurani MJ, Satiani B, Go MR (2017). Common carotid artery end-diastolic velocity and acceleration time can predict degree of internal carotid artery stenosis. J Vasc Surg.

[17] Wang S, Wang Y, Gao M, Tan Y (2022). Acceleration time to ejection time ratio in fetal pulmonary artery system can predict neonatal respiratory disorders in gestational diabetic mellitus women. Clin Hemorheol Microcirc.

[18] Maréchaux Sylvestre, Tribouilloy C (2021). Acceleration time in aortic stenosis: a new life for an old parameter. Circ Cardiovasc Imaging.

[19] Gruber M, Sommerset J, Hansen-Nyholm C, Feliciano B, Vea Y, Karmy-Jones R (2023). Potential utility of hand acceleration time in quantifying the degree of malperfusion in patients with suspected hemodialysis access–induced distal ischemia. J Vasc Ultrasound.

[20] Sommerset J, Sapru AB, Teso D, Karmy-Jones R (2020). Arterial duplex and palmar acceleration time in an upper extremity trauma case. J Vasc Ultrasound.

[21] Sommerset J, Sapru AB, Teso D, Karmy-Jones R (2021). A possible role for arterial duplex and hand acceleration time in diagnosing and managing shock. J Vasc Ultrasound.

[22] Fontaine R, Kim M, Kieny R (1954). Surgical treatment of peripheral circulation disorders. Helv Chir Acta.

[23] Gonzalo B, Videla S, Moranas J, Carnaval T, Herranz C, Espinar E, Iborra E (2024). Hand acceleration time is a valuable ultrasonographic tool in hand perfusion as adjuvant evaluation for diagnosing chronic upper limb ischaemia. Ann Vasc Surg.

[24] Mann HB, Whitney DR (1947). On a test of whether one of two random variables is stochastically larger than the other. Ann Math Statist.

